# Development and validation of a prediction model of catheter-related thrombosis in patients with cancer undergoing chemotherapy based on ultrasonography results and clinical information

**DOI:** 10.1007/s11239-022-02693-7

**Published:** 2022-08-16

**Authors:** Shanhong Lin, Ning Zhu, YihanZhang, Liping Du, Shengmin Zhang

**Affiliations:** 1 Department of Ultrasound, Ningbo First Hospital, 59 Liuting Road, Ningbo, 315010 Zhejiang Province People’s Republic of China; 2 Department of Respiratory and Critical Care Medicine, Ningbo First Hospital, Ningbo, People’s Republic of China

**Keywords:** Cancer, Chemotherapy, Catheters, Thrombosis, Nomogram, Prediction

## Abstract

Central venous catheters can be used conveniently to deliver medications and improve comfort in patients with cancer. However, they can cause major complications. The current study aimed to develop and validate an individualized nomogram for early prediction of the risk of catheter-related thrombosis (CRT) in patients with cancer receiving chemotherapy. In total, 647 patients were included in the analysis. They were randomly assigned to the training (n = 431) and validation (n = 216) cohorts. A nomogram for predicting the risk of CRT in the training cohort was developed based on logistic regression analysis results. The accuracy and discriminatory ability of the model were determined using area under the receiver operating characteristic curve (AUROC) values and calibration plots. Multivariate logistic regression analysis showed that body mass index, risk of cancer-related thrombosis, d-dimer level, and blood flow velocity were independent risk factors of CRT. The calibration plot showed an acceptable agreement between the predicted and actual probabilities of CRT. The AUROC values of the nomogram were 0.757 (95% confidence interval: 0.717–0.809) and 0.761 (95% confidence interval: 0.701–0.821) for the training and validation cohorts, respectively. Our model presents a novel, user-friendly tool for predicting the risk of CRT in patients with cancer receiving chemotherapy. Moreover, it can contribute to clinical decision-making.

## Introduction

Cancer is the second leading cause of mortality worldwide [1], with approximately 18.1 million new cases recorded in 2018 [2]. In China, 4.3 million new cases of cancer were reported in the same year [3]. Although cancer treatment has significantly improved, chemotherapy is still the preferred treatment option. Further, adjunctive therapy is recommended for several types of malignant tumors. Chemotherapeutic drugs are commonly delivered via venous access devices into the central vein. Thus, a reliable central venous access is essential. Ultrasound-guided central venous catheterization (CVC) is associated with high success rates and low mechanical complication rates. Moreover, it is the gold standard technique [4] and has long been strongly recommended for auxiliary CVC [5–8], thereby providing a simple and reliable infusion route for chemotherapy. Cancer is associated with a highly thrombogenic environment, which increases the risk of venous thromboembolism (VTE). Some epidemiological studies have shown that 4–7% of patients with cancer who are hospitalized develop VTE [9, 10]. Hence, they are at seven-fold higher risk for complications than those without cancer [11]. This risk is further compounded by the presence of a CVC device. In a previous study, approximately 5% of patients with cancer who developed catheter-related thrombosis (CRT) are symptomatic. Meanwhile, 14–18% are asymptomatic during treatment [12].

Patients with cancer who develop venous thrombosis commonly have poor prognoses and low survival rates. Khorana et al. [10] examined the causes of mortality in 4466 patients with cancer at 117 centers in the US. Results showed that thrombotic events were a major factor in non-cancer-related mortality (9.2%), with 33% of deaths caused by VTE. CRT is a major complication of CVC, which impairs the patency of the central venous lumen and catheter performance, ultimately leading to catheter dysfunction [13, 14]. This phenomenon, in turn, disrupts treatment plans and increases the cost of care in patients with cancer [15, 16]. Moreover, thrombosis treatment may be associated with a high risk of bleeding. CRT is a severe complication common among patients with malignant tumors, and early identification of patients who are at high risk of this complication is essential. Nevertheless, most patients with CRT are asymptomatic. Occasionally, CRT is detected relatively late and cannot be managed effectively, leading to poor clinical outcomes. Therefore, a simple, reliable, and accurate method for assessing the individual risk of CRT is essential in patients with malignant tumors.

A nomogram is an intuitive graphical prediction model that allows precise individualized risk predictions [17]. Several studies have shown that a nomogram has a good predictive ability for disease risk in patients with malignant tumors receiving chemotherapy and those with postoperative venous thrombosis [18–21]. However, there are only few studies on the prediction of CRT in patients with malignant tumor. In addition, previous reports did not focus on the vascular ultrasound-related features of CRT. A prospective cohort study found that local factors were more likely to be associated with peripherally inserted central CRT than systemic factors [22]. However, it did not assess factors correlated with centrally inserted central catheters (CICCs). Our model focused on local factors associated with vascular ultrasound characteristics, in addition to the main factors correlated with cancer. The current study aimed to establish and validate a nomogram prediction model for CRT using variables that can be easily assessed and routinely collected before catheterization in patients receiving chemotherapy for malignant tumor. The model could allow clinicians to identify patients who are at high risk for CRT and to perform examinations in a timely manner to improve diagnosis and management.

## Methods

### Patient selection

This retrospective study included patients with cancer who initially received chemotherapy via CVC at the Ningbo First Hospital from January 2019 to December 2020. Data of these patients were collected from the hospital’s clinical database. This research was approved by the ethics committee of Ningbo First Hospital (Approval Number: 2021RS126). Written informed consent was obtained from each patient before central venous catheter placement.

The inclusion criteria were as follows: (1) patients aged ≥ 18 years, (2) those with active cancer with definite pathological diagnosis, (3) those receiving systemic chemotherapy via ultrasound-guided CICC catheterization, and (4) those who underwent follow-up ultrasound until CICC removal or the development of intravenous thrombosis. The exclusion criteria were as follows: (1) patients with VTE or CRT confirmed via precannulation examination, (2) those who received long-term anticoagulation therapy before catheterization due to a history of thrombosis, (3) those with a previous history of hematological diseases, (4) those with an expected survival time of < 1 month, (5) those with incomplete case information, and (6) those who were lost to follow-up.

Finally, 647 patients with cancer were included in the study. Using a randomized 2:1 classification scheme, 431 patients were assigned to the training cohort for establishing a predictive model. Meanwhile, 216 patients were included in the validation cohort for evaluating the model’s performance.

### CVC placement and nursing care

CICCs are placed by interventionists under ultrasound guidance. Only patients with ultrasound-guided CICCs inserted via the left or right subclavian, jugular, or femoral veins were included in this review. Patients with peripherally inserted central venous catheter (PICC) were excluded.

All patients underwent venipuncture under real-time ultrasound guidance with the single-lumen 6-Fr CVC using the modified Seldinger technique. The specific insertion vein and location were determined via ultrasonography. The internal jugular vein was most preferred, followed by the subclavian and femoral veins. Before extubation during hospitalization, all CVC lumens were flushed daily with 10 mL of normal saline and 5 mL of heparin to ensure lumen patency.

### Data collection

Before ultrasound-guided catheter placement, prospective ultrasound measurements were performed, and the diameter of the indwelling vein and the maximum blood flow velocity were recorded. Before examination, patients were instructed to rest for 30 min to stabilize respiratory and hemodynamic status. During ultrasonography, all patients were placed in the supine position with their limbs naturally extended. Blood flow velocity was assessed if the spectral curve had a stable waveform.

The endpoint was CRT confirmed via ultrasonography. In this study, the analysis was limited to the development of thrombi at the insertion site. Ultrasonography could be performed any time if patients developed clinical symptoms of CRT (e.g., pain, swelling, tenderness, and congestion) at the site of CVC placement. Otherwise, ultrasonography was performed to rule out thrombus formation before catheter removal.

Screening for asymptomatic thrombosis is not routinely performed in clinical settings. Thus, in this study, it was not conducted before extubation due to practicality reasons. Data on the characteristics of patients and laboratory examination results were obtained from the electronic medical record system of our institution. These included (but not limited to) general information (age, sex, and body mass index [BMI]), previous medical history (including surgery, previous chemotherapy [CT], catheterization, and concomitant diseases such as deep venous thrombosis/pulmonary embolism, hyperlipidemia, hypertension, diabetes mellitus, cerebrovascular disease and other heart diseases, nephrosis, chronic obstructive pulmonary disease, and bacteremia), cancer-related characteristics (risk of thrombosis, cancer stage), laboratory parameters (d-dimer and fibrinogen levels, platelet count), treatment information (radiotherapy, parenteral nutrition, and anti-infective and antiplatelet treatment), and catheter-related characteristics (insertion side and vein, blood flow velocity, and catheter-to-vein ratio). Table 1 shows all 36 variables.

**Table 1:** Baseline characteristics of patients with a CRT in training cohort and validation cohort

Variables	Total sample (n = 647)	Training cohort (n = 431)	Validation cohort (n = 216)	P-value
CRT, n (%)	244 (37.7%)	166 (38.5%)	78 (36.1%)	0.552
Demographic characteristics				
Age (years, mean ± SD)	60.71 ± 8.59	60.50 ± 8.34	61.12 ± 9.07	0.386
Sex				0.579
Male	359 (55.5%)	236 (54.8%)	123 (56.9%)	
Female	288 (44.5%)	195 (45.2%)	93 (43.1%)	
BMI (mean ± SD)	23.97 ± 3.86	23.92 ± 3.24	24.08 ± 4.9	0.612
Patient-related characteristics				
Medical history				
Smoking	181 (28.0%)	122 (28.3%)	59 (27.3%)	0.818
Surgery	101 (15.6%)	71 (16.5%)	30 (13.9%)	0.393
Previous CT	98 (15.1%)	70 (16.2%)	28 (13.0%)	0.273
Previous catheter	123 (19.0%)	86 (20.0%)	37 (17.1%)	0.388
Prior DVT/PE	35 (5.4%)	23 (5.3%)	12 (5.6%)	0.908
Comorbidities				
Hyperlipidemia	60 (9.3%)	41 (9.5%)	19 (8.8%)	0.767
Hypertension	64 (9.9%)	44 (10.2%)	20 (9.3%)	0.703
Diabetes mellitus	60 (9.3%)	39 (9.0%)	21 (9.7%)	0.781
CVD	17 (2.6%)	12 (2.8%)	5 (2.3%)	0.725
Heart disease	22 (3.4%)	14 (3.2%)	8 (3.7%)	0.763
Nephrosis	11 (1.7%)	8 (1.9%)	3 (1.4%)	0.759
COPD	15 (2.3%)	9 (2.1%)	6 (2.8%)	0.583
Bacteremia	3 (0.5%)	2 (0.5%)	0	0.555
Cancer-related characteristics				
Cancer thrombosis risk				0.240
Low or intermediate	185 (28.6%)	120 (27.8%)	65 (24.9%)	
High	381 (58.9%)	251 (58.2%)	130 (60.2%)	
Very high	81 (12.5%)	60 (13.9%)	21 (9.7%)	
Stage of cancer				0.717
Localized tumor (stages I–III)	519 (80.2%)	344 (79.8%)	175 (81.0%)	
Advanced tumor (stage IV)	128 (19.8%)	87 (20.2%)	41 (19.0%)	
Laboratory parameters (mean ± SD)				
d-Dimer (mg/L)	0.65 ± 0.45	0.63 ± 0.41	0.71 ± 0.52	0.052
Platelet count (× 10^9^/L)	264 ± 153	259 ± 142	273 ± 174	0.299
Fibrinogen (g/L)	3.97 ± 0.77	3.95 ± 0.73	4.01 ± 0.83	0.337
Treatments				
Radiotherapy	19 (2.9%)	13 (3.0%)	6 (2.8%)	0.866
Parenteral nutrition	47 (7.3%)	35 (8.1%)	12 (5.6%)	0.236
Anti-infective therapy	47 (7.3%)	30 (7.0%)	17 (7.9%)	0.674
Antiplatelet treatment	17 (2.6%)	11 (2.6%)	6 (2.8%)	0.866
Catheter-related characteristics				
Insertion side of catheter				0.681
Left	27 (4.2%)	17 (3.9%)	10 (4.6%)	
Right	620 (95.8%)	414 (96.1%)	206 (93.4%)	
Insertion vein				0.803
Subclavian vein	126 (19.5%)	81 (18.8%)	45 (20.8%)	
Jugular vein	503 (77.7%)	340 (78.9%)	163 (75.5%)	
Femoral vein	18 (2.8%)	10 (2.3%)	8 (3.7%)	
Blood flow velocity (cm/s, mean ± SD)	24.38 ± 8.75	24.63 ± 8.55	23.89 ± 9.12	0.312
Catheter-to-vein ratio (mean ± SD)	0.18 ± 0.03	0.18 ± 0.03	0.18 ± 0.04	0.072

*BMI* Body mass index, *CT* chemotherapy, *DVT* deep venous thrombosis, *PE* pulmonary embolism, *CVD* cerebrovascular disease, *COPD* chronic obstructive pulmonary disease

Based on previous research [23–25], he cancer types were divided into three categories according to the risk of thrombosis: extremely high risk (gastric, pancreatic, hematological, and brain cancer, mesothelioma, and cancer of unknown primary), high risk (lung, gynecologic, and genitourinary excluding prostate cancer), and low risk (breast, colorectal, prostate, bone, head and neck, cutaneous, and testicular cancer and melanoma). Hematological cancers were also categorized, with solid tumors associated with an extremely high risk of thrombosis based on a previous research [24].

### Statistical analysis

Descriptive data were expressed as number (percentage). Parametric data were presented as mean (+ standard deviation) and nonparametric data as median (range). The Student’s *t*-test or the Mann–Whitney *U* test was performed to compare continuous variables, and categorical variables were compared using the chi-square (χ^2^) test or the Fisher’s exact test.

Univariate and multivariate logistic regression analyses were performed. To identify the significant independent risk factors of CRT, variables that were statistically significant in the univariate logistic regression analysis were included in the multivariate logistic regression analyses. Based on the results of the multivariate logistic regression analysis, a nomogram was constructed to assess the risk of CRT, and the model was discriminated and corrected. The model was validated using the bootstrap method with 1000 resamples, and the area under the receiver operating characteristic curve (AUROC) was calculated as a measure of discrimination. To gauge the predictive accuracy the nomogram, the observed and predicted probabilities were plotted against each other. Validation was conducted using validation cohort patients, and the discriminative ability and predictive accuracy performance of the model were assessed using AUROC and calibration plots.

All statistical analyses were performed using R (P < 0.05). The nomogram prediction model was developed using the RMS package of R (r4.1.3). Internal verification was performed using Bootstrap, and C-index was calculated using the RMS software package. The calibration curve was obtained by comparing bias-corrected predictions and observations.

## Results

### Clinical and ultrasound characteristics

Table 1 presents the clinical and ultrasound characteristics of the patients in the training and validation cohorts. There were no significant differences between the training and validation cohorts in terms of basic clinical characteristics, laboratory parameters, and vascular ultrasound characteristics (P > 0.05) (Table 1). The incidence rates of CRT did not significantly differ between the training and validation groups [38.8% (n = 251) vs. 39.3% (n = 85)].

### CRT in the training and validation cohorts

A total of 244 patients with CRT (37.7%) were included in our study, and 34.4% (84/244) of these were symptomatic and 65.6% (160/244) were asymptomatic with a mean catheterization time of 13.2 ± 8.8 days. The majority of cases of symptomatic thrombus (n = 65; 77.4%) occurred within 1 week after insertion. Detailed information on catheter-associated thrombi in the training and validation cohorts is presented in Table 2.

**Table 2:** CRT in the training and validation cohorts

Catheter-related thrombi	Total sample (n = 244)	Training cohort (n = 166)	Validation cohort (n = 78)
Catheter days (days, mean ± SD)	13.2 ± 8.8	13.3 ± 9.0	12.9 ± 8.5
Symptomatic thrombosis time (days)			
0–7 days	65 (77.4%)	47 (79.7%)	18 (72.0%)
7–14 days	13 (15.5%)	8 (13.6%)	5 (20.0%)
> 14 days	6 (7.1%)	4 (6.8%)	2 (8.0%)
Asymptomatic thrombosis	160 (65.6%)	107 (64.5%)	53 (67.9%)
Symptomatic thrombosis	84 (34.4%)	59 (35.5%)	25 (32.1%)

### Baseline characteristics of the training cohort

Compared with patients with non-CRT, those with CRT were older (P = 0.004, Table 3). Further, they had a higher BMI (P < 0.001, Table 3), d-dimer level (P < 0.001, Table 3), and cancer thrombosis risk (P < 0.001, Table 3) but lower catheter-to-vein ratio (P < 0.001, Table 3) and blood flow velocity (P = 0.019, Table 3).

**Table 3:** Baseline characteristics of patients with CRT and Non-CRT in training cohort

Variables	Training cohort (n = 431)		
	Non-CRT (n = 265)	CRT (n = 166)	P-value
Demographic characteristics			
Age (years, mean ± SD)	59.58 ± 7.98	61.96 ± 8.88	0.004
Sex			0.859
Male	146 (55.1%)	90 (54.2%)	
female	119 (44.9%)	76 (45.8%)	
BMI (mean ± SD)	23.31 ± 3.32	24.89 ± 2.86	< 0.001
patient-related characteristics			
Medical history			
Smoking	67 (25.3%)	55 (33.1%)	0.078
Surgery	44 (16.6%)	27 (16.3%)	0.926
Previous CT	41 (15.5%)	29 (17.5%)	0.584
Previous catheter	52 (19.6%)	34 (20.5%)	0.828
Prior DVT/PE	15 (5.7%)	8 (4.8%)	0.705
Comorbidities			
Hyperlipidemia	23 (8.7%)	18 (10.8%)	0.456
Hypertension	31 (11.7%)	13 (7.8%)	0.197
Diabetes mellitus	23 (8.7%)	16 (9.6%)	0.735
CVD	9 (3.4%)	3 (1.8%)	0.385
Heart disease	10 (3.8%)	4 (2.4%)	0.437
Nephrosis	5 (1.9%)	3 (1.8%)	0.994
COPD	6 (2.3%)	3 (1.8%)	0.992
Bacteremia	2 (0.8%)	0	0.525
Cancer-related characteristics			
Cancer thrombosis risk			0.002
Low or intermediate	98 (37.0%)	22 (13.3%)	
High	141 (53.2%)	110 (66.3%)	
Very high	26 (9.8%)	34 (20.5%)	
Stage of cancer			0.174
Localized tumor (stages I–III)	206 (77.7%)	138 (83.1%)	
Advanced tumor (stage IV)	59 (22.3%)	28 (16.9%)	
Laboratory parameters			
d-Dimer (mg/L)	0.49 ± 0.34	0.84 ± 0.42	< 0.001
Platelet count (× 10^9^/L)	257 ± 149	260 ± 128	0.831
Fibrinogen (g/L)	3.91 ± 0.75	4.02 ± 0.71	0.132
Treatments			
Radiotherapy	10 (3.8%)	3 (1.8%)	0.245
Parenteral nutrition	24 (9.1%)	11 (6.6%)	0.369
Anti-infective therapy	21 (7.9%)	9 (5.4%)	0.320
Antiplatelet treatment	9 (3.4%)	2 (1.2%)	0.160
Catheter-related characteristics			
Insertion side of catheter			0.781
Left	11 (4.2%)	6 (3.6%)	
Right	254 (95.8%)	160 (96.4%)	
Insertion vein			0.873
Subclavian vein	49 (18.5%)	32 (1.9%)	
Jugular vein	210 (79.2%)	130 (7.8%)	
Femoral vein	6 (2.3%)	4 (2.4%)	
Previous catheterization			
Blood flow velocity (cm/s, mean ± SD)	25.39 ± 8.29	23.41 ± 8.85	0.019
Catheter-to-vein ratio (mean ± SD)	0.16 ± 0.03	0.20 ± 0.02	< 0.001

*BMI* Body mass index, *CT* chemotherapy, *DVT* deep venous thrombosis, *PE* pulmonary embolism, *CVD* cerebrovascular disease, *COPD* chronic obstructive pulmonary disease

### Feature selection

Based on the univariate analysis of feature selection in the training cohort, age, BMI, d-dimer level, cancer-related thrombosis risk, and blood flow velocity were significantly associated with early CRT after CVC (Table 4). Via multivariate analyses, we screened for the significant predictive factors of CRT. Results showed that BMI, d-dimer level, cancer-related thrombosis risk, and blood flow velocity were independent risk factors of CRT after CVC in patients with malignant tumors receiving chemotherapy (Table 4).

**Table 4:** Univariate and multivariate analysis of the associations between CRT and baseline characteristics in training cohort

Variables	Univariate analysis				Multivariate analysis			
	β	OR	95% CI	P-value	β	OR	95% CI	P-value
Demographic characteristics								
Age (years, mean ± SD)	0.035	1.035	1.011–1.060	0.004	0.021	1.021	0.991–1.052	0.170
BMI (mean ± SD)	0.160	1.173	1.099–1.252	< 0.001	0.151	1.163	1.083–1.249	< 0.001
Cancer-related characteristics								
Cancer thrombosis risk								
Low or intermediate	Ref				Ref			
High	1.263	3.536	2.091–5.978	< 0.001	1.296	3.655	62.088–6.397	< 0.001
Very high	1.772	5.885	2.956–11.716	< 0.001	1.983	7.263	3.421–15.418	< 0.001
Stage of cancer								
Localized tumor (stages I–III)	Ref							
Advanced tumor (stage IV)	− 0.345	0.708	0.430–1.167	0.176				
Laboratory parameters (mean ± SD)								
d-Dimer (mg/L)	1.304	3.684	2.173–6.246	< 0.001	1.157	3.18	1.839–5.499	< 0.001
Platelet count (× 10^9^/L)	0.464	1.590	1.060–2.385	0.025				
Fibrinogen (g/L)	0.089	1.093	0.825–1.449	0.536				
Catheter-related characteristics								
Insertion side of catheter								
Left	Ref							
Right	0.044	1.045	0.665–1.641	0.849				
Insertion vein								
Subclavian vein	Ref							
Jugular vein	− 0.053	0.948	0.577–1.557	0.833				
Femoral vein	0.021	1.021	0.267–3.904	0.976				
Blood flow velocity (cm/s,mean ± SD)	− 0.032	0.968	0.944–0.993	0.011	− 0.038	0.963	0.937–0.990	0.008
Catheter-to-vein ratio (mean ± SD)	− 0.341	0.711	0.448–1.127	0.147				

*BMI* Body mass index, *CT* chemotherapy, *DVT* deep venous thrombosis, *PE* pulmonary embolism, *CVD* cerebrovascular disease, *COPD* chronic obstructive pulmonary disease, β is the regression coefficient, *CI* confidence interval, *OR* odds ratio

### Construction of the nomogram for predicting CRT

**Fig.1:**
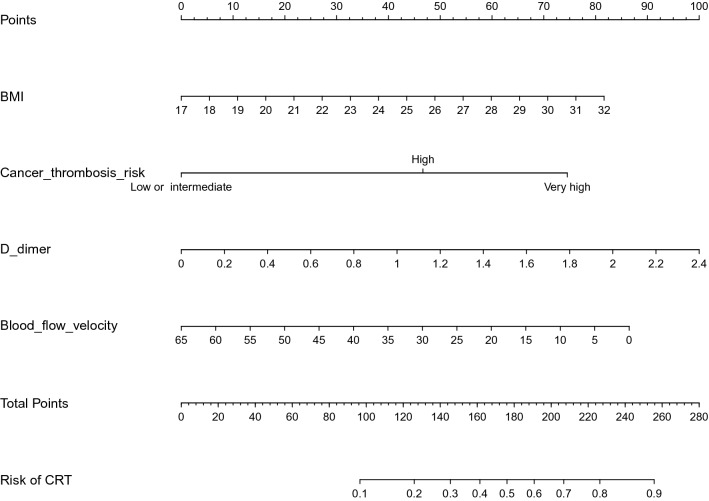
Nomogram predicting the risk of CRT in patients with cancer udergoing chemotherapy. For all patients, adding up the points identified on the points scale for all four indicators. The sum is then placed on the “Total Point” axis. Finally, the risk of CRT can be determined by the probability of “CRT” corresponding to “Total Points”

Based on four independent predictors of CRT determined via multivariate logistic regression analysis, the nomogram for predicting CRT in patients with malignant tumors receiving chemotherapy was established (Fig. 1).

### Accuracy of the nomogram

**Fig.2:**
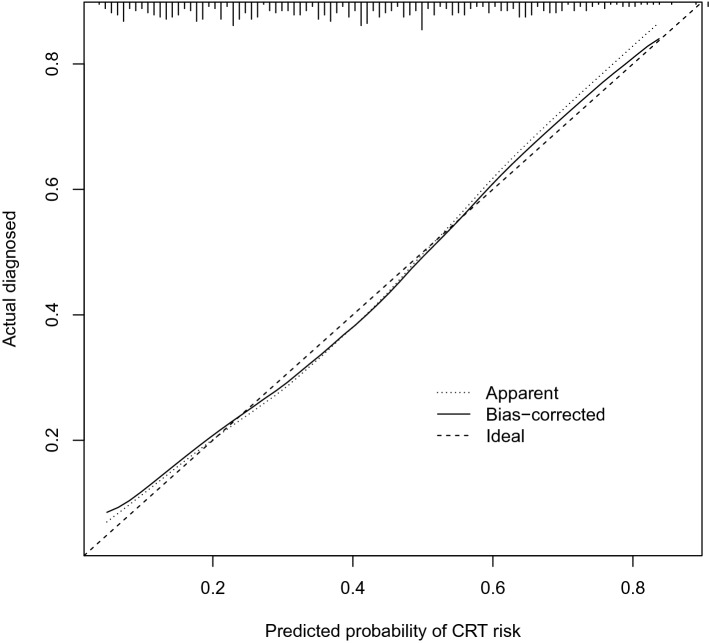
Calibration plot of the nomogram for the probability of CRT in patients with cancer udergoing chemotherapy (bootstrap 1000 repetitions)

**Fig.3:**
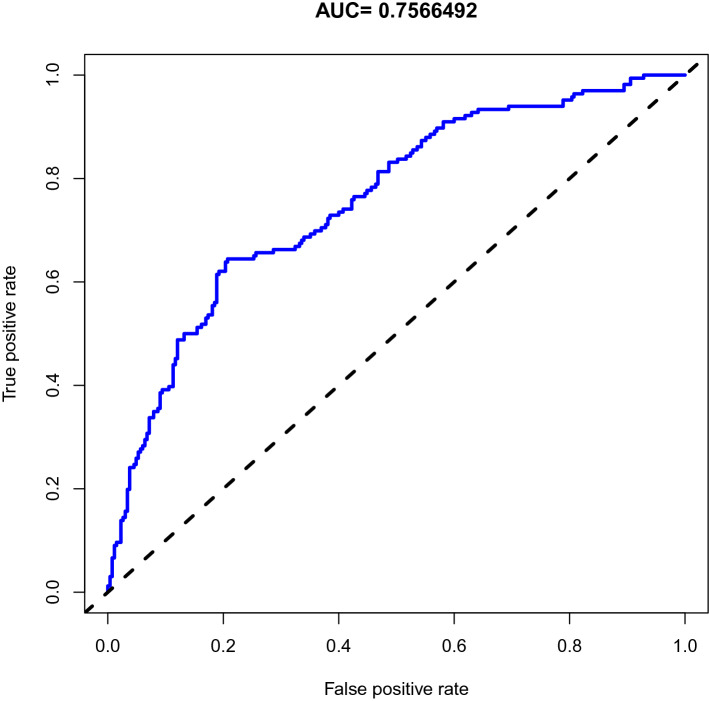
Area under the curve of the nomogram model of CRT in patients with cancer udergoing chemotherapy

The calibration curve of the nomogram predication model had a good consistency in the training pairs (Fig. 2). The AUC of the nomogram calculated using R was 0.757 (95% CI 0.717–0.809, Fig. 3). Thus, the model had good discriminative abilities.

**Fig.4:**
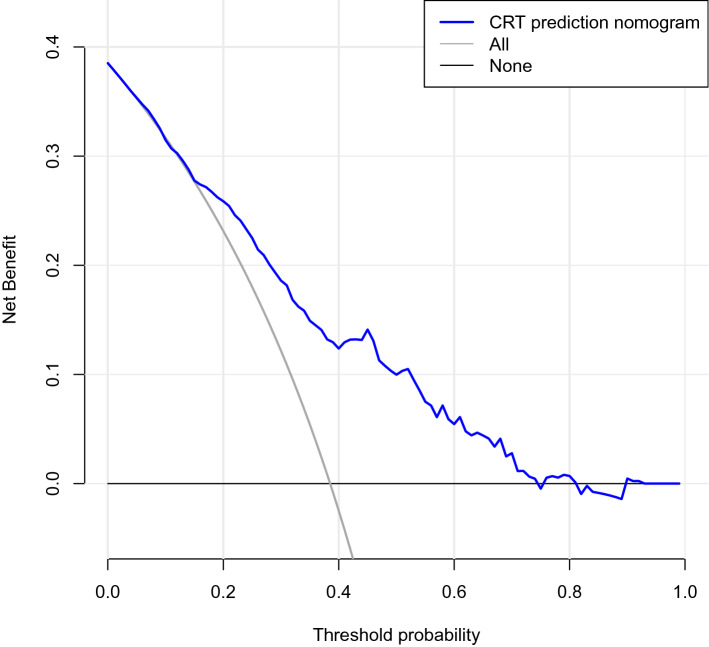
Decision curve analysis of the nomogram of CRT in patients with cancer udergoing chemotherapy. The blue line represents the CRT risk nomogram, with the y-axis measuring net benefit. The thin solid line indicates that CRT occurs during catheterization in all patients. The thick solid line (parallel to the x-axis) represents the assumption that no patients developed CRT. The net benefit was calculated by subtracting the proportion of all patients who are false positive from the proportion who are true positive, weighting by the relative harm of forgoing treatment compared with the negative consequences of an unnecessary treatment. In this study, 6% (the intersection of blue line and thin solid line) was false positive rate and 70% (the intersection of blue line and thick solid line) was false negative rate

According to the decision curve analysis, the predictive model had a greater net benefit within a threshold probability interval of 6%–70% (Fig. 4). In addition, the predicted and actual probabilities of each patient in the validation cohorts were compared. The AUROC of the predicted model was 0.761 (95% CI 0.701–0.821).

## Discussion

Chemotherapy and the use of central venous access are essential in most patients with cancer. Chemotherapy is considered an independent risk factor of VTE [26, 27]. Some risk factors associated with CRT among patients receiving chemotherapy have been reported in the literature. However, a specific scoring system for classifying patients based on risk has not been developed, which is not conducive for individualized prediction. Previous studies have shown numerous risk factors correlated with CRT. However, their results were controversial, thereby making it difficult to establish predictive models of CRT.

Nomograms are important in modern medical decision-making models because of their high accuracy and good discriminative capability [21, 28, 29]. At present, several models are being used to predict VTE in patients with cancer [21, 23, 28, 30]. However, these models did not consider the risk factors associated with vascular ultrasound features. The blood flow velocity and catheter-to-vein ratio were added to our nomogram model that was specifically designed to predict the risk of CRT in patients undergoing chemotherapy.

The current study included patients with CICCs inserted under ultrasonography guidance. Ultrasound-guided surgery reduces the effect of technical factors compared with blind puncture. Moreover, pretube testing can identify asymptomatic CRT. To the best of our knowledge, this nomogram is the first model of CICC-associated thrombosis in patients receiving chemotherapy via ultrasound-guided catheterization.

We focused on variables that can be easily assessed and routinely collected before initiating catheterization for chemotherapy. The current study established and validated a simple clinical model based on clinical and vascular ultrasound-related features for predicting the development of CRT in patients receiving chemotherapy for malignant tumors. The nomogram prediction model identified four clinical and vascular-related features, which were BMI, cancer thrombosis risk, d-dimer level, and blood flow velocity. The model had good discrimination ability in both the training and validation cohorts. The model can be easily used in clinical settings and can effectively help clinicians identify patients receiving chemotherapy who are at high risk of CRT at an early time. As a result, patients can undergo ultrasound examinations and receive treatment in a timely manner.

There was an extremely wide variation in the incidence of CRT (ranging from 12 to 74%) among different types of cancer. The apparent differences in incidence rates might be attributed to variations in patient selection and the types of venous thrombosis examined in these studies. Our research included patients who received chemotherapy during the study period, those with active cancer, and those at high risk of venous thrombosis. A more important reason could be that our criteria for the definition of thrombus were relatively lenient. That is, they included all types of thrombi, such as fibrin sheaths, that could be detected via ultrasonography. Blood flow velocity was an independent predictor of CRT, and this result was consistent with that of previous studies. Hemodynamically, the distribution of blood in the vessels and the speed of blood flow can be used to determine whether there is a need to declare an individual antithrombotic state [23]. Previous studies have shown that a smaller venipuncture diameter and a larger catheter-to-vein ratio are strongly associated with CRT [22, 31]. However, our study did not obtain a similar result. This might be correlated with differences in vein puncturing. In a previous study, PICC was used, and the peripheral veins with smaller vein diameters were selected.

Previous studies have shown that a catheter diameter-to-vein diameter ratio of > 0.35 was significantly correlated with CRT (OR: 1.689; 95% CI 1.023–2.789) [31]. However, the central veins with relatively large diameters, such as the internal jugular vein, were used on our patients. Ultrasound-guided placement was conducive for selecting puncture sites with large diameters. The ratio of catheter veins was < 0.35, which could not reflect the influence of this difference. This might also be a reason why several studies have found a higher risk of thrombosis with the use of PICCs.

Consistent with previous studies, this study showed an association between BMI and d-dimer level and the incidence of CRT. d-dimer is an effective marker of coagulation and fibrinolysis activation [32]. Previous studies have shown that patients with malignant tumors have elevated d-dimer levels, thereby increasing the risk of thrombosis by 4 times [33]. Arpaia et al. [34] showed that the prechemotherapy d-dimer level of patients with malignant tumors can be used as an independent indicator of venous thrombosis.

A BMI of > 25 kg/m^2^ is independently associated with a higher risk of CRT [35–37]. Overweight and obesity can increase the risk of thrombosis by three-fold [36, 38]. Catheterization can be difficult in patients with obesity, and repeated punctures can damage the vessel wall, thereby increasing the risk of thrombosis. Catheterization may be challenging in patients with obesity, and repeated punctures are associated with a high risk of thrombosis.

Previous studies have shown that the Khorana score can be used to predict the risk of VTE in patients with cancer. A development and validation study of two independent prospective cohorts found that only the tumor-site risk category was significantly associated with the risk of VTE according to the Khorana score [21]. Similarly, in our study, cancer-related thrombosis was significantly correlated with CRT. Our study again supports the finding that a higher risk of cancer thrombosis was associated with an increased risk of CRT. Surprisingly, tumor stage was not correlated with thrombosis; a possible reason for this is that the participants were more exposed to additional risk factors or that the effect could have been masked by subsequent disease progression.

The univariate analysis showed an association between the risk of CRT and age. However, it was not considered an independent risk factor. In a previous study, BMI, platelet count, and hemoglobin level were associated with VTE [23]. However, they were not risk factors of VTE in our study. Diabetes, high blood pressure, and smoking, which are indicators of chronic cardiovascular disease, were not considered as indicators of CRT. One possible explanation is that primary tumor and local catheter factors are more likely to influence the development of CRT.

Our study had several limitations. First, it was a single-center research and could not reflect the whole image of patients with cancer receiving chemotherapy. Although the study had a prospective design, the results of the retrospective analysis might have been biased or incomplete. For example, data retrieved from the clinical database did not include the possible predictors of CRT that were not routinely detected in clinical practice but had been previously observed. Further, the laboratory parameters were not dynamically evaluated. Third, some patients with asymptomatic CRT might have gone undetected because routine imaging was not performed. Nevertheless, multicenter prospective studies must be provided to further confirm the reliability of the model.

This study developed and validated a nomogram with good accuracy and discriminativeness. Thus, it can facilitate individualized prediction of CRT in patients receiving chemotherapy. Further, the model can be applied effectively in daily clinical practice and can identify patients receiving chemotherapy who are at high risk of CRT. Further, it can help clinicians in decision-making and implementing efficient individualized preventive treatment based on the specific risk of each patient.
